# The unmet drug-related needs of patients with diabetes in Ethiopia: a systematic review and meta-analysis

**DOI:** 10.3389/fendo.2024.1399944

**Published:** 2024-05-30

**Authors:** Mengistie Yirsaw Gobezie, Nuhamin Alemayehu Tesfaye, Tewodros Solomon, Mulat Belete Demessie, Teklehaimanot Fentie Wendie, Yaschilal Muche Belayneh, Assefa Mulu Baye, Minimize Hassen

**Affiliations:** ^1^ Department of Clinical Pharmacy, School of Pharmacy, College of Medicine and Health Sciences, Wollo University, Dessie, Ethiopia; ^2^ Department of Pharmacology, School of Pharmacy, College of Medicine and Health Sciences, Wollo University, Dessie, Ethiopia; ^3^ School of Pharmacy, College of Health Science Addis Ababa University, Addis Ababa, Ethiopia

**Keywords:** diabetes mellitus, unmet drug related needs, drug-related problems, systematic review, meta analysis, Ethiopia

## Abstract

**Background:**

Diabetes is a major health concern globally and in Ethiopia. Ensuring optimal diabetes management through minimizing drug therapy problems is important for improving patient outcomes. However, data on the prevalence and factors associated with unmet drug-related needs in patients with diabetes in Ethiopia is limited. This systematic review and meta-analysis aims to provide a comprehensive analysis of the prevalence of unmet drug-related needs among patients with diabetes mellitus in Ethiopia.

**Methods:**

A thorough exploration of databases, including PubMed, Scopus, Hinari, and Embase and Google Scholar, was conducted to identify pertinent studies. Inclusion criteria involved observational studies that reported the prevalence of unmet drug-related needs in Ethiopian patients with diabetes. The quality of the studies was assessed using Joanna Briggs Institute (JBI) checklists. A random-effects meta-analysis was employed to amalgamate data on study characteristics and prevalence estimates, followed by subsequent subgroup and sensitivity analyses. Graphical and statistical assessments were employed to evaluate publication bias.

**Results:**

Analysis of twelve studies involving 4,017 patients revealed a pooled prevalence of unmet drug-related needs at 74% (95% CI 63-83%). On average, each patient had 1.45 unmet drug-related needs. The most prevalent type of unmet need was ineffective drug therapy, 35% (95% CI 20-50). Type 2 diabetes, retrospective study designs, and studies from the Harari Region were associated with a higher prevalence. Frequently reported factors associated with the unmet drug-related needs includes multiple comorbidities, older age, and polypharmacy. Notably, the results indicated significant heterogeneity (I^2 ^= 99.0%; p value < 0.001), and Egger’s regression test revealed publication bias with p<0.001.

**Conclusion:**

The prevalence of unmet drug-related needs among diabetes patients with diabetes in Ethiopia is high with the most prevalent issue being ineffective drug therapy. Targeted interventions are needed; especially patients on multiple medications, advanced age, with comorbidities, and prolonged illness duration to improve diabetes management and outcomes.

**Systematic review registration:**

https://www.crd.york.ac.uk/prospero, identifier CRD42024501096.

## Introduction

Diabetes is a metabolic disorder characterized by hyperglycemia due to defects in insulin secretion and/or action, causing long-term organ damage, and thus requires proper management to control the disease state and slow down the progression towards microvascular and macrovascular complications (1). It is one of the most challenging public health problems in the 21^st^ century being one of four priority noncommunicable diseases targeted for action by world leaders. Approximately 537 million adults, constituting 10.5% of the global adult population, are currently living with diabetes, reflecting a prevalence of 1 in 10 individuals. Projections indicate that this number is anticipated to increase to 643 million by the year 2030 and further escalate to 783 million by 2045 (2).majority of it being type 2 diabetes. Over the past decade, diabetes prevalence has risen faster in low- and middle-income countries than in high-income countries (3).

Effective diabetes management involves appropriate dietary intake, exercise, and adhering to necessary medication regimens. Large, population-based, prospective trials have shown that intensive glucose management can dramatically decrease the rate of microvascular complications in diabetes mellitus as well as protect against macrovascular complications to some degree (4, 5). According to the American Diabetes Association recommendation, a reasonable glycosylated hemoglobin (HbA1c) goal for many nonpregnant adults is <7% (6).

High HbA1c levels have consistently proven to be associated with increased risk of morbidity and mortality in long-term studies (7–10). Poorly controlled diabetes increases the risk of heart attack, stroke, kidney failure, leg amputation, vision loss, nerve damage, and dying prematurely. Its complications are resulting in increasing disability, reduced life expectancy and enormous health costs for virtually every society through direct medical costs and loss of work and wages (3, 10–12).

Despite this evidence and the many advances in diabetes care, 33–49% of patients still do not meet targets for glycemic, blood pressure, or cholesterol control (6, 13, 14). This is the case in Africa where a large number of people with diabetes do not reach the recommended HbA1c targets; likely due to a limited access to adequate health services, low diabetic knowledge, and a high prevalence of drug therapy problems (15–18).

Drug therapy problems are a consequence of a patient’s drug-related needs that have gone unmet. A drug therapy problem is any undesirable event experienced by a patient that involves, or is suspected to involve drug therapy, and that interferes with achieving the desired goals of therapy (19). It is central to pharmaceutical care practice (19–22). Drug therapy problems may lead to increased morbidity, mortality, healthcare costs, and recurrent hospital admissions and prolonged hospitalization (23). A systematic review indicated that drug-related problems account for more than 15.4% of hospital admissions and 2.7% death rate (24).

Patients with diabetes are at risk of drug therapy problems as they are receiving multiple medications, which can happen at any step during the treatment process and it affects the therapeutic outcome (17, 25). Studies done in Eastern Ethiopia and South-East Ethiopia reported that 64.2% and 88% of the study participants had at least one drug therapy problem with poor glycemic control (26, 27). An average of 1.8 drug therapy problems per patient were identified in Eastern Ethiopia (25).

A proactive approach to prevent, identify and resolve drug therapy problems is required in order to realize the best possible outcomes from drug therapy. Involvement of clinical pharmacists as a member of the healthcare team helps in identification and prevention of clinically significant drug therapy problems (28–30). In Brazil, pharmacist intervention increases compliance, reduces drug therapy problems and, consequently, improves glycemic control among patients with type 2 diabetes (31). In India, clinical pharmacists had a crucial role in minimizing drug related problems among diabetic patients with co-existing hypertension (1). In Addis Ababa Ethiopia, provision of medication therapy management services had a potential to reduce drug therapy problems and improve the clinical parameters (32).

The prevalence of drug therapy problems and associated factors varies between different populations ranging from 42% to 99.5% (25, 33); thus, a comprehensive data showing the magnitude of the problem and identifying specific risk factors is urgently needed for targeting interventions most effectively. Therefore, the aim of this review is to investigate the pooled prevalence of drug therapy problems and contributing factors among patients with diabetes mellitus.

## Methods

### Review protocol

The results of this review were reported in accordance with the Preferred Reporting Items for Systematic Reviews and Meta-analysis (PRISMA) guideline (34). The protocol for this systematic review and meta-analysis has been registered in the Prospero database under the registration number PROSPERO 2024: CRD42024501096.

### Databases and search strategy

An inclusive literature search was conducted to retrieve studies reporting the unmet drug related need of patients with diabetes in Ethiopia. Both electronic and gray literature searches were carried out systematically. We used different electronic bibliographic databases like PubMed, Google Scholar, Wiley Online Library, Hinari(research4life), Embase, Scopus and, Web of Sciences. Our search included studies written in the English language. In addition, the proceedings of professional associations, and universities repository were screened. A direct Google search was also conducted using the bibliographies of the identified studies to include additional relevant studies omitted during electronic database searches.

The search was conducted using MeSH terms, and combined key terms are taken from the review question. All potentially eligible studies were accessed by using the following combination keys; “Drug therapy problems”, “drug-related problems”, “Unmet drug-related need”, “Medication-related problems”, “Drug-related errors”, “Pharmacotherapy problems”, “Medication errors”, “Prescription errors”, **“**DM”, “Diabetes patients”, “Diabetic patients”, “Diabetes mellitus patients”, “Type2 diabetes mellitus patients”, “Type 1 diabetes mellitus patients “and Ethiopia. The search strings were employed using “AND” and “OR” Boolean operators. Endnote 20 (35) was used to manage references and remove duplicates.

### Inclusion and exclusion criteria

The search encompassed studies published prior to the search date (10 January 2024). Studies were considered eligible if they met the following inclusion criteria: [I] clearly defined drug therapy problems and accurately reports the prevalence of unmet drug related need of patients with diabetes; [II] designed as observational studies, including cross-sectional studies, surveillance studies; [III] conducted in Ethiopia; and [IV] published in the English language. Exclusion criteria comprised case reports, case-control studies, reviews, commentaries, editorials, and conference abstracts, which were not actively sought during the search process.

### Eligibility and quality assessment

To eliminate duplicate studies, we utilized Endnote version 20 (35) as our reference manager. Two authors independently scrutinized the titles and abstracts to identify articles for further consideration in the full-text review. The full text of the remaining articles was then obtained, and two investigators independently conducted eligibility assessments and subsequently evaluated the quality of the studies using the JBI critical appraisal checklist designed for studies reporting prevalence data (36).

The JBI critical appraisal checklist encompassed various criteria, including [I] the appropriateness of the sampling frame to address the target population; [II] the suitability of the study participant sampling technique and the adequacy of the sample size; [III] a detailed description of study subjects and setting; [IV] a thorough analysis of the data and the validity and reliability of methods used for measuring the prevalence and types of unmet drug-related needs; and [V] the appropriateness of statistical analyses and the adequacy of the sample size. Discrepancies were resolved through consensus. Studies scoring five or above out of nine criteria were categorized as low-risk in terms of methodological quality.

### Data extraction

Data extraction was carried out by three authors following a predefined data extraction format. In instances of discrepancies, a repeated procedure was employed to ensure accuracy and consistency. Another three authors performed the consolidation and summarization of the final set of articles that met our inclusion criteria. These authors compiled comprehensive tables containing information on authors, study period, publication year, study design, setting, region, sample size, study population, number of unmet drug-related needs, and types of unmet drug-related needs and risk of bias.

### Outcome of interest

**Figure 1:**
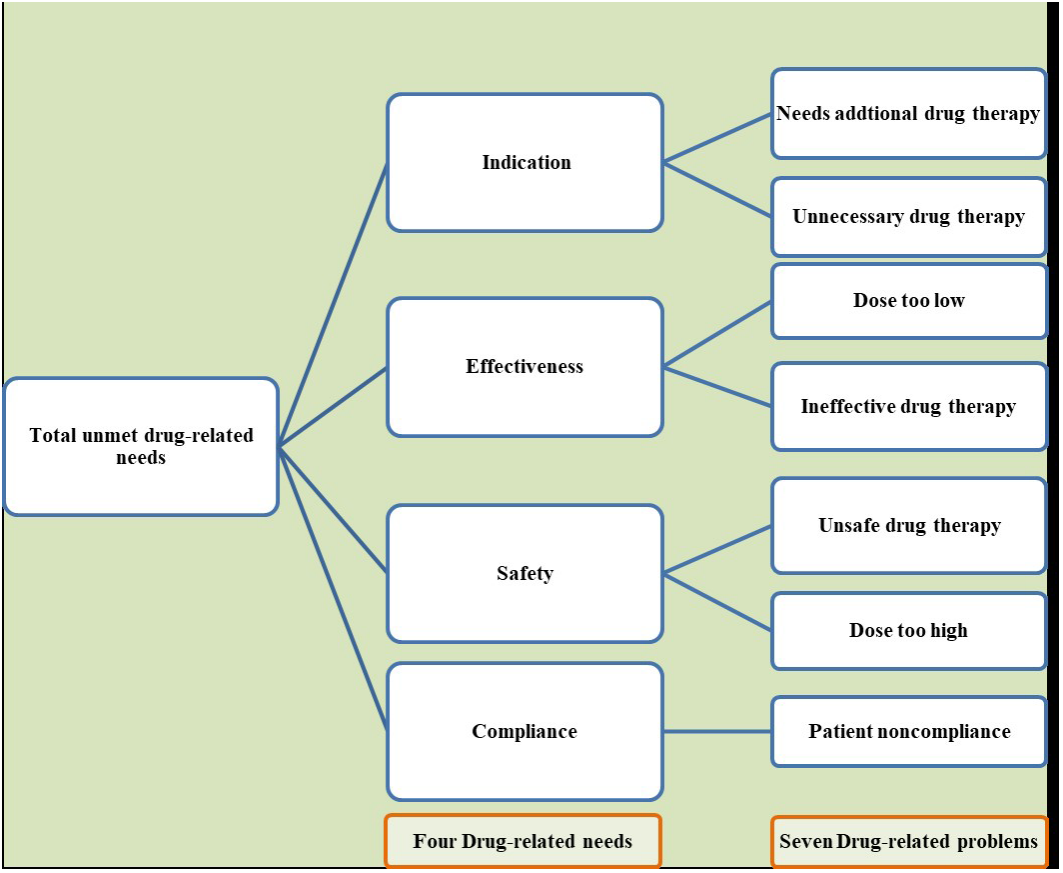
Classification of total unmet drug-related needs into four overarching themes and their corresponding seven drug-related problems.

The primary focus of this meta-analysis is the assessment of unmet drug-related needs, calculated by dividing the total number of patients experiencing at least one drug-related problem by the total number of participants in the study. These problems are categorized into four main areas of drug-related needs: indication, safety, effectiveness, and compliance, then these four drug-related needs are further subdivided into seven distinct classes of drug therapy problems. All the included primary studies used the Pharmaceutical Care Network Europe Association(PCNE) (37) for the classification of drug-related problems This classification is depicted in **Figure 1**.

### Data analysis

To estimate the prevalence of unmet drug-related needs of DM patients in Ethiopia, we employed a weighted inverse variance random-effects model (38). Addressing variations in the pooled prevalence estimates, we conducted subgroup analyses based on the types of DM, regions where the studies were conducted and the study designs employed for assessment of unmet drug-related needs. Heterogeneity among the studies was thoroughly examined using a forest plot, meta-regression, and the I^2^ statistic, with 25%, 50%, and 75% denoting low, moderate, and high heterogeneity, respectively (39). A significance level for the Q test with a p-value less than 0.05 was used as an indicator of heterogeneity.

The presentation of results was facilitated through a comprehensive forest plot. To evaluate the potential presence of publication bias, a Funnel plot and Egger’s regression test were employed, where a p-value less than 0.05 in Egger’s test suggested significant publication bias. Additionally, Trim and fill analysis were conducted as a supplementary measure to assess publication bias (40).

Ensuring the stability of the summary estimate, a sensitivity analysis was conducted by systematically omitting individual studies. This analysis aimed to gauge the impact of each study on the overall estimate, providing insights into the robustness of the meta-analysis. The entire meta-analysis was conducted using STATA version 17 (41) a widely recognized statistical software, to ensure precision and reliability in the analysis.

## Result

### Characteristics of included studies

**Figure 2:**
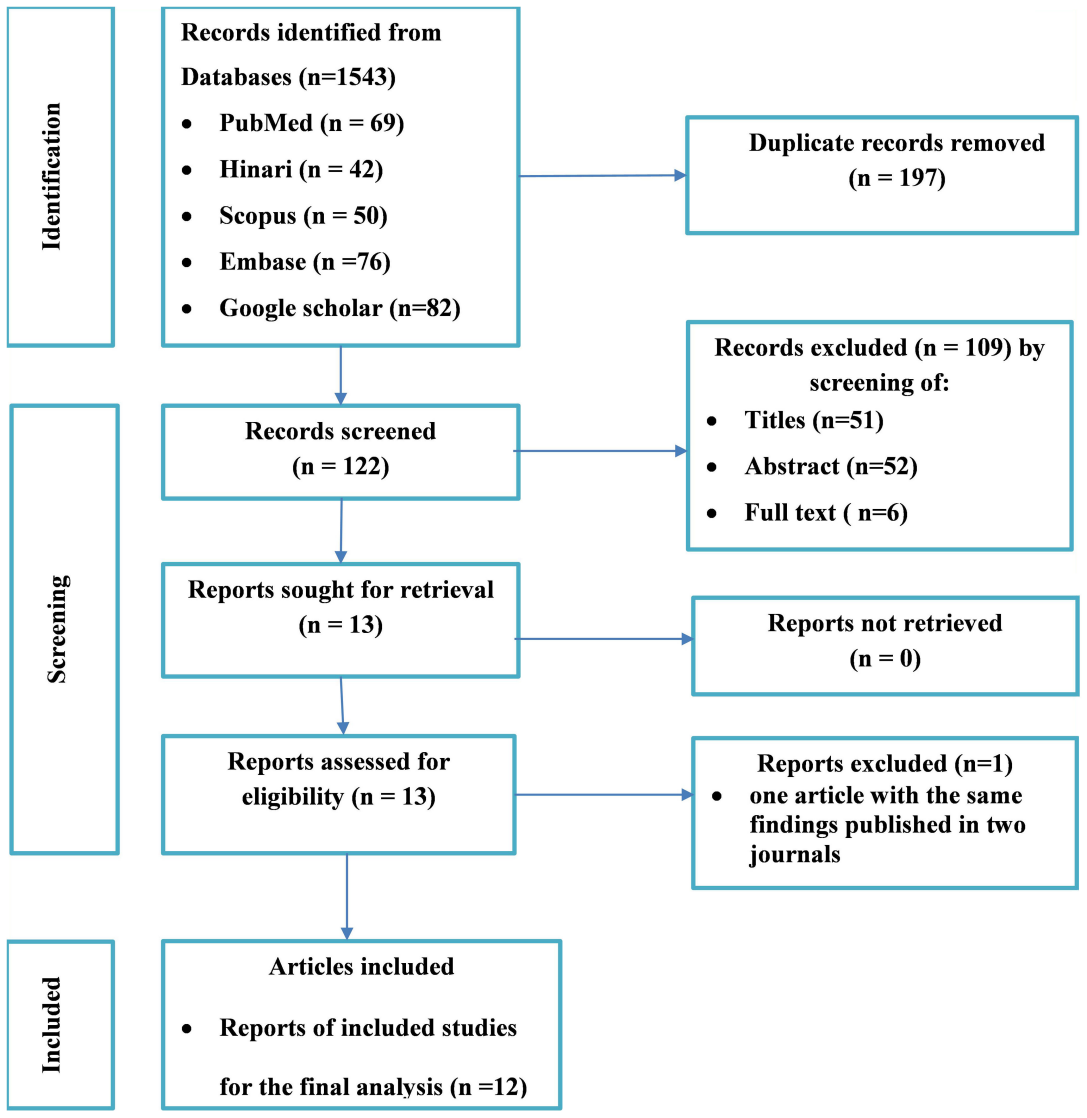
Flow diagram of the included studies for the systematic review and meta-analysis of the prevalence of the unmet drug-related needs of patients with diabetes in Ethiopia.

A total of 319 potential studies were identified through various sources, including 69 articles from PubMed, 42 articles from Hinari (research4life), 76 articles from EMBASE, 50 articles from Scopus, and 82 articles from google scholar. The outcomes of the search, along with the reasons for exclusion during the study selection process, are illustrated in **Figure 2**. After thorough scrutiny, 12 articles were deemed suitable for inclusion in the meta-analysis, focusing on assessing the prevalence of unmet drug-related need of DM patients in Ethiopia. All included studies adhered to either cross-sectional or cohort study designs. Among these, eight studies specifically investigated the prevalence of unmet drug-related needs in type-2 DM patients (25, 26, 42–47), while the remaining studies were analyzed on both type-1 and type-2 DM patients (33, 48–50). For a detailed overview of the included studies, including their characteristics, refer to **Table 1**.

**Table 1:** General characteristics of included studies for systematic review and meta-analysis.

Authors	Study Period	Regions	Study Design	Study Setting	Study population	Sample Size	Mean DRN	Pts with DRN	Total-DRN	Risks of Bias
**Koyra et al**	2015	SNNPR	RCS	Hospital	T2DM	243	1.8 ± 0.751	202	378	Low
**Yimama et al**	2016	Oromia	CSP	Hospital	T2DM	300	1.65 ± 1.05	246	494	Low
Ayele et al	2017	Harar	CS	Hospital	T2DM	203	1.8	202	364	Low
**Abdulmalik et al**	2018	Harar	RCS	Hospital	T2DM	148	0.9	95	127	Low
**Argaw AM,et al**	2017	Oromia	CS	Hospital	T2DM	216	2.06 ± 0.861	191	446	Low
**Demoz et al**	2017	Addis Ababa	CS	Hospital	All	418	1.16 ± 0.42	177	207	Low
**Mechessa et al**	2019	SNNPR	CS	Hospital	All	141	1.10 ± 0.44	82	156	Low
**Kefale et al**	2019	Amhara	CS	Hospital	T2DM	423	1.86 ± 0.53	264	491	Low
**Sheleme et al**	2019	Oromia	CS	Hospital	All	330	1.38 ± 0.85	279	455	Low
**Belayneh et al**	2019	Amhara	RCS	Hospitals&HC	T2DM	156	1.18	126	149	Low
**Negash et al**	2019	Addis Ababa	CS	Hospital	All	409	1.94 ± 1.06	298	578	Low
**Kahssay et al.**	2022	SNNPR	CS	Hospital	T2DM	117	1.47	83	172	Low

DRN, Drug-Related Need; SNNPR, Southern nation nationalities and people region; RCS, Retrospective cross sectional; CS, Cross Sectional; Pts, Patients.

### Quality of the included studies

Every study underwent evaluation using the JBI critical appraisal checklist designed for studies reporting prevalence data. The application of JBI quality appraisal checklists revealed that none of the included studies were deemed of poor quality, and as a result, none were excluded from the meta-analysis (**Supplementary File**).

### Meta-analysis

#### Prevalence of unmet drug-related needs of DM patients in Ethiopia

**Figure 3:**
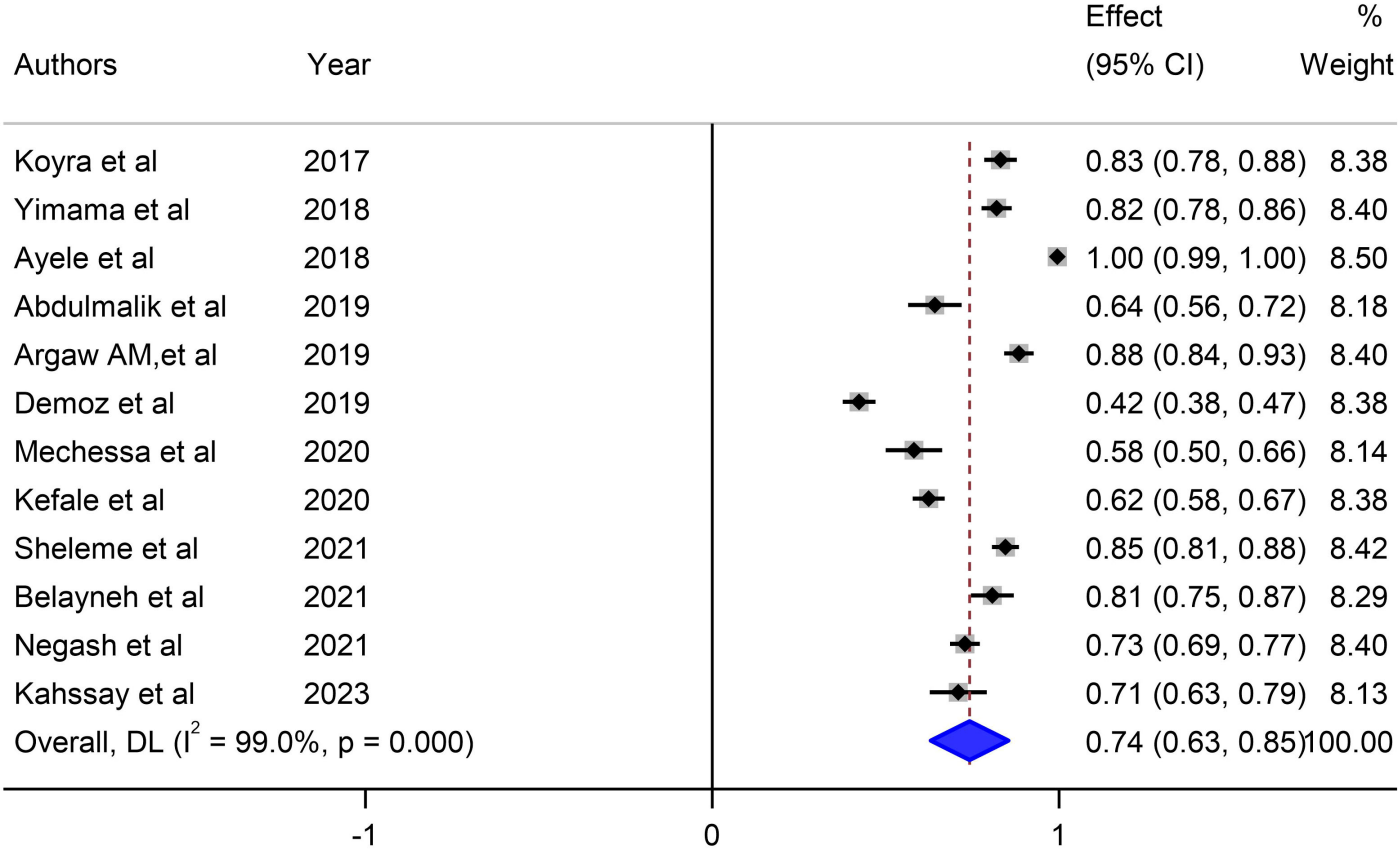
Pooled prevalence of unmet drug-related needs of DM patients in Ethiopia.

Our meta-analysis sought to comprehensively assess the prevalence of unmet drug-related needs of DM patients in Ethiopia, synthesizing data from multiple studies to derive a pooled estimate. The pooled prevalence of unmet drug-related needs of DM patients in Ethiopia was 74% (95% CI: 63, 83, I^2^ = 99%; p value < 0.001) (**Figure 3**).

#### Mean number of unmet drug-related needs per patient in DM patients in Ethiopia

**Figure 4:**
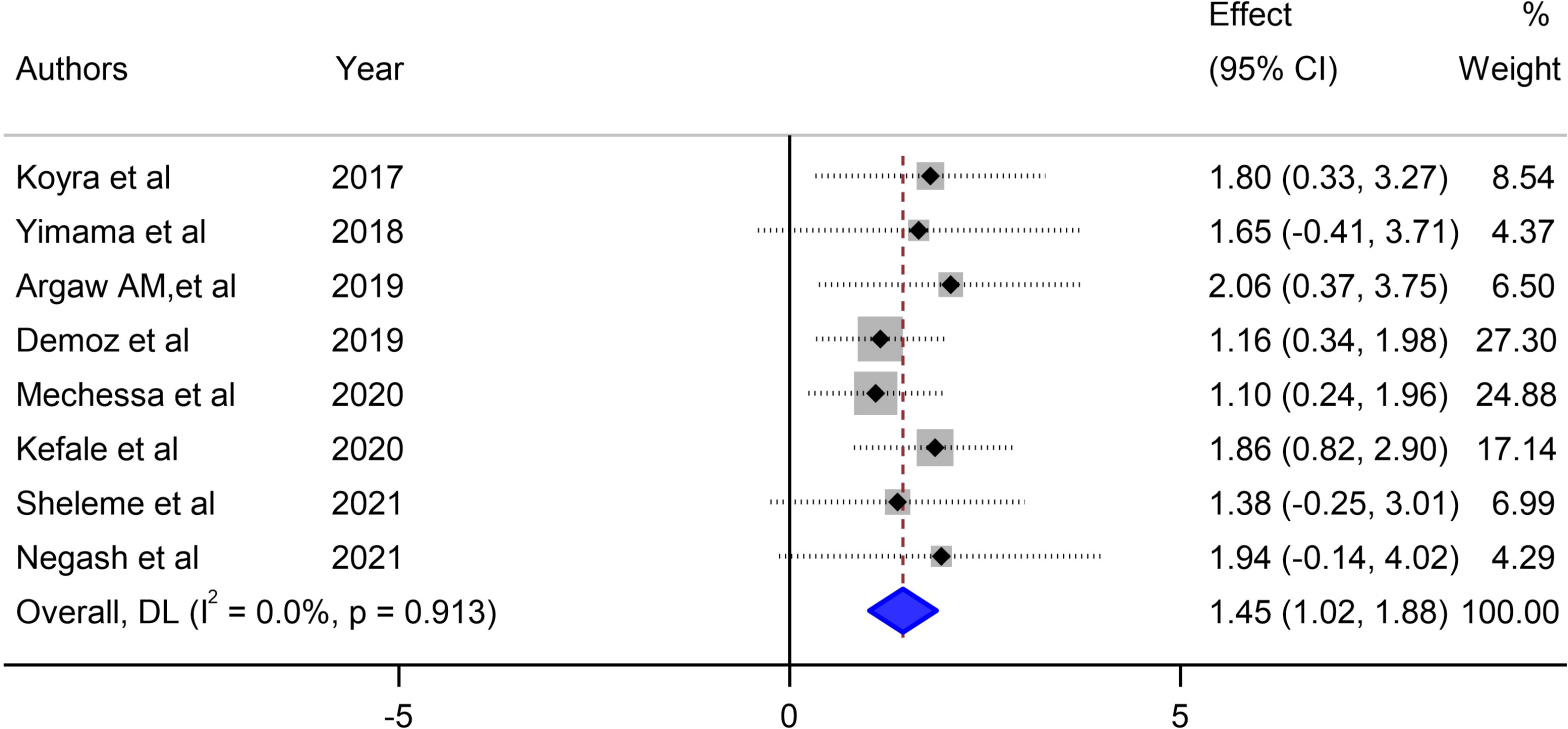
Pooled mean number of unmet drug-related needs per patient in diabetes mellitus patients in Ethiopia.

Our meta-analysis assessed the mean number of unmet drug-related needs per patient in diabetic patients in Ethiopia and synthesized data from 8 studies that reported this outcome to derive a pooled estimate. The pooled mean of unmet drug-related needs per patient in patients with DM in Ethiopia was 1.45 (95% CI: 1.02–1.88, I^2^ = 0.0%; p value < 0.913) (**Figure 4**).

#### Types of unmet drug-related needs in DM patients

A comprehensive analysis was conducted, categorizing unmet drug-related needs into four main themes and seven specific drug-related problems. Patients requiring additional drug therapy showed a significant prevalence of 25% (95%CI: 14, 36). Conversely, patients undergoing high-dose drug therapy exhibited a lower prevalence of 4% (95% CI: 2, 5), as shown in **Table 2**. This highlights notable differences in prevalence among various unmet drug-related needs and their corresponding distinct drug-related problems.

**Table 2:** Pooled prevalence of each drug-related problem derived from the four categories of unmet drug-related needs.

Types of Unmet Drug-Related Needs	Types of Drug-related Problems	Events in Each Category	Total Events	Pooled Estimate (95%CI)	I^2^	p-value
Indication	Needs additional drug therapy	1043	4017	25(14,36)	99.3	<001
	Unnecessary drug therapy	297	4017	8(6,10)	89.7	<001
Effectiveness	Ineffective drug therapy	855	4017	19(11,27)	99.5	<001
	Dosage too low	610	4017	15(9,21)	98.4	<001
Safety	Adverse drug reaction	433	4017	9(5,13)	96.9	<001
	Dosage too high	171	4017	4(2,5)	94.2	<001
Compliance	Noncompliance	607	3439	16(11,20)	98.8	<001

### Subgroup analysis

**Figure 5:**
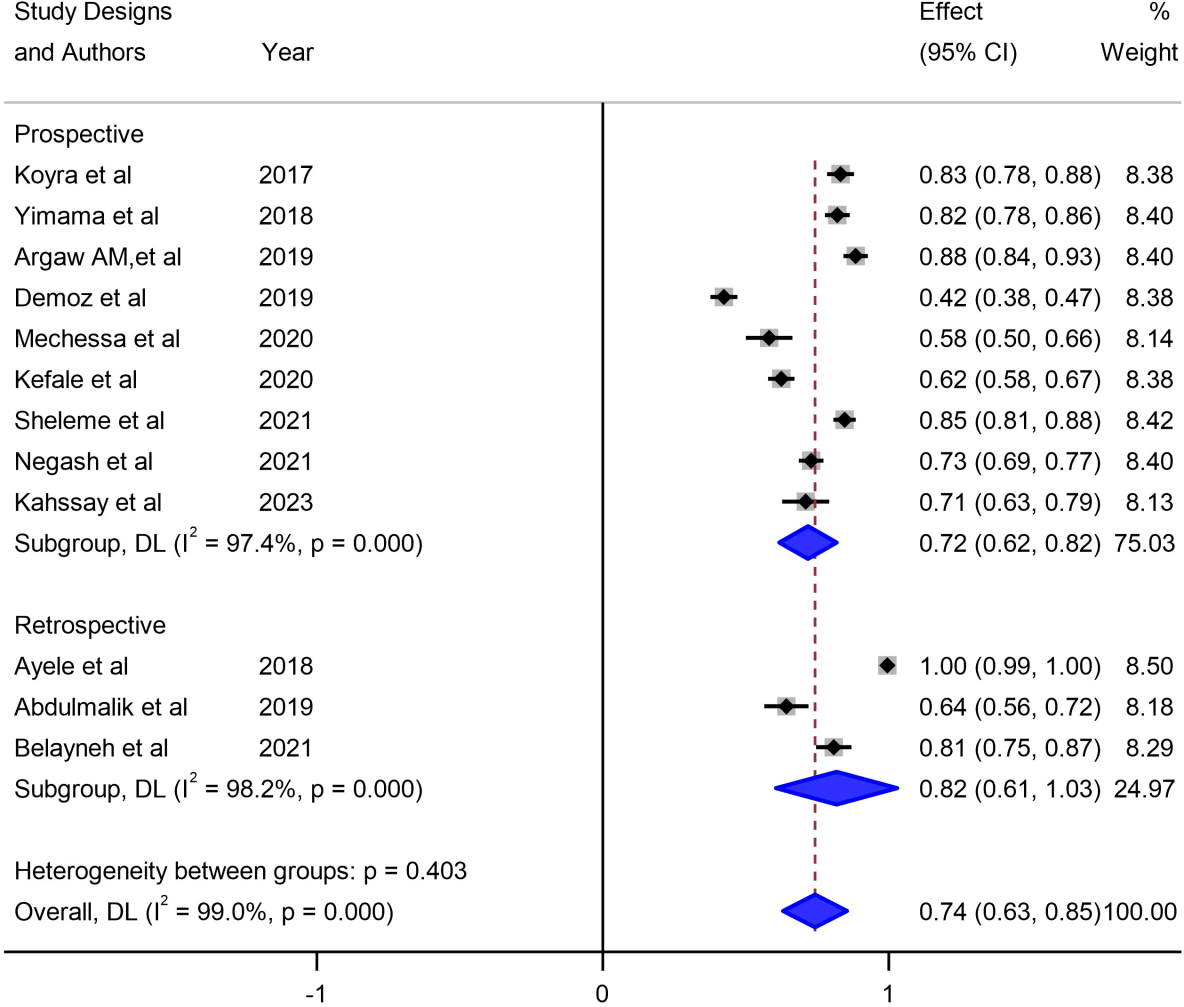
Subgroup analysis of unmet drug-related needs of DM patients in Ethiopia based on study design.

**Figure 6:**
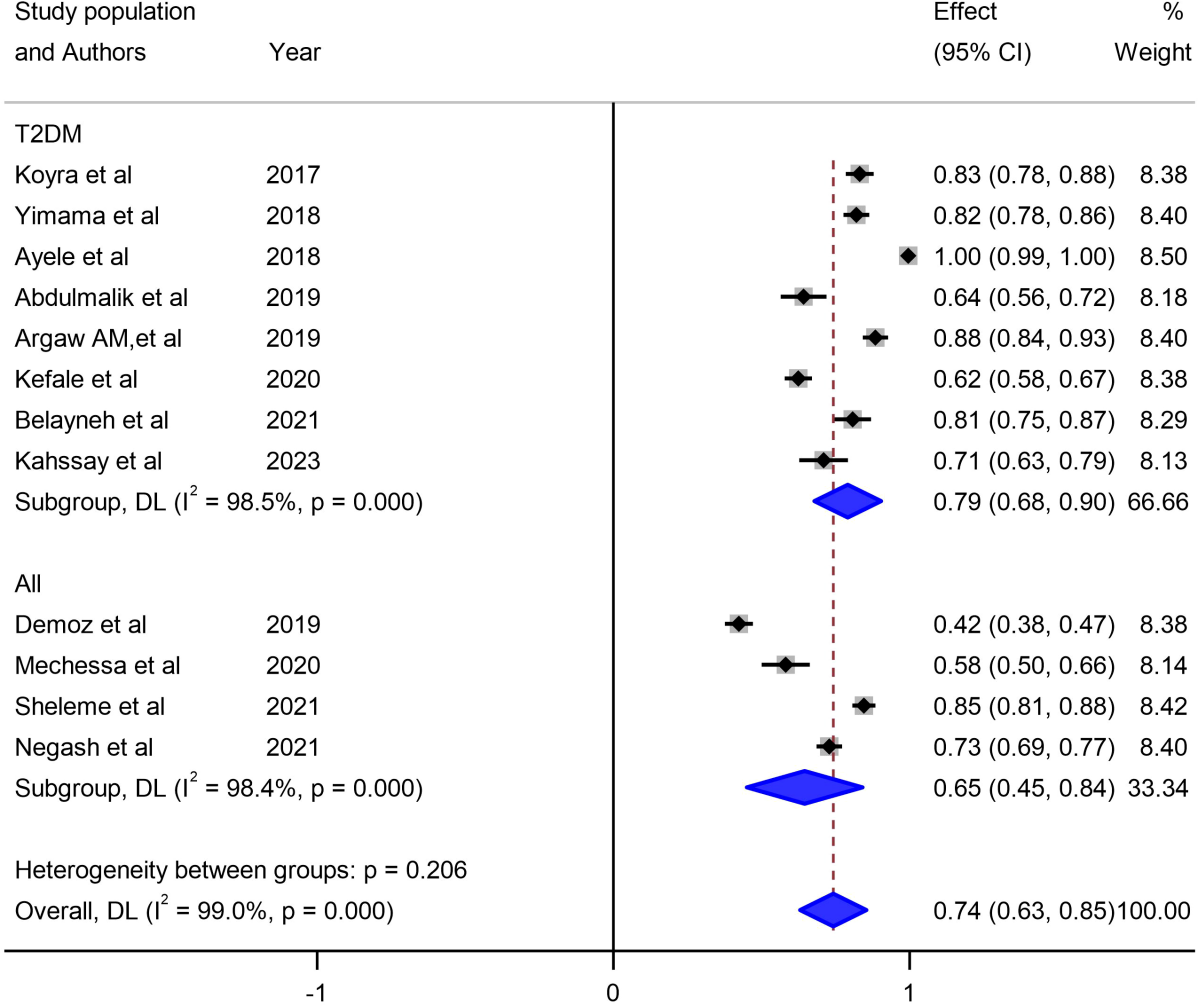
Subgroup analysis of unmet drug-related needs of DM patients in Ethiopia based on types of DM.

**Figure 7:**
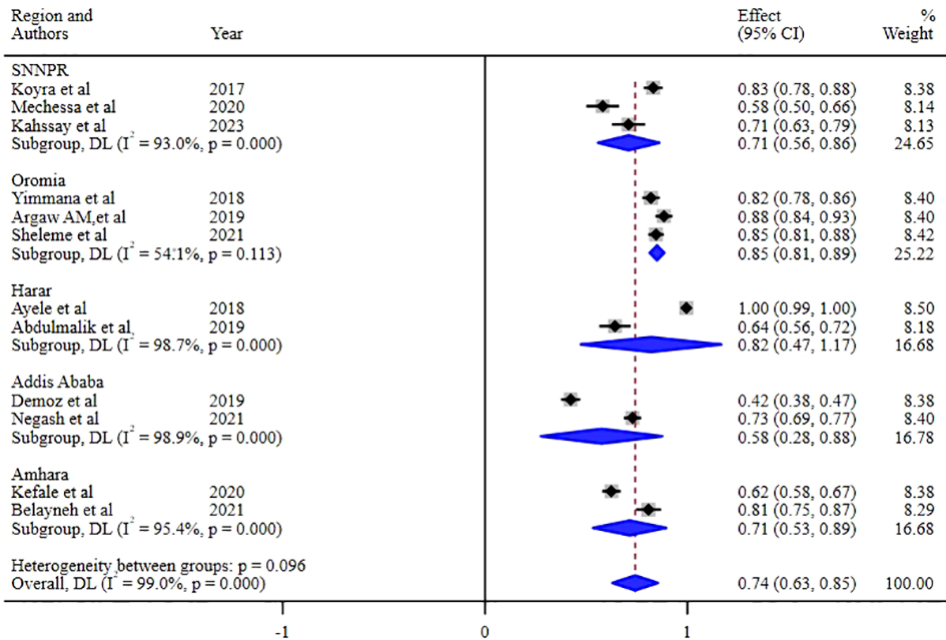
Subgroup analysis of the unmet drug-related needs of DM patients in Ethiopia based on the regions where studies were conducted.

Our investigation into understanding the origin of heterogeneity involved conducting subgroup analysis based on the types of diabetes mellitus (DM), study design, and specific study setting. The results indicated a higher prevalence of 79% (95% CI: 68, 90) in type-2 DM patients, 82% (95% CI: 61, 103) in studies with a retrospective design, and 82% in studies conducted at Harar as illustrated in **Figures 5**, **6** and **7**. The lowest prevalence (58%) of unmet drug-related need amidst diabetic patients is reported in Addis Ababa, the capital of Ethiopia.

### Heterogeneity analysis

**Figure 8:**
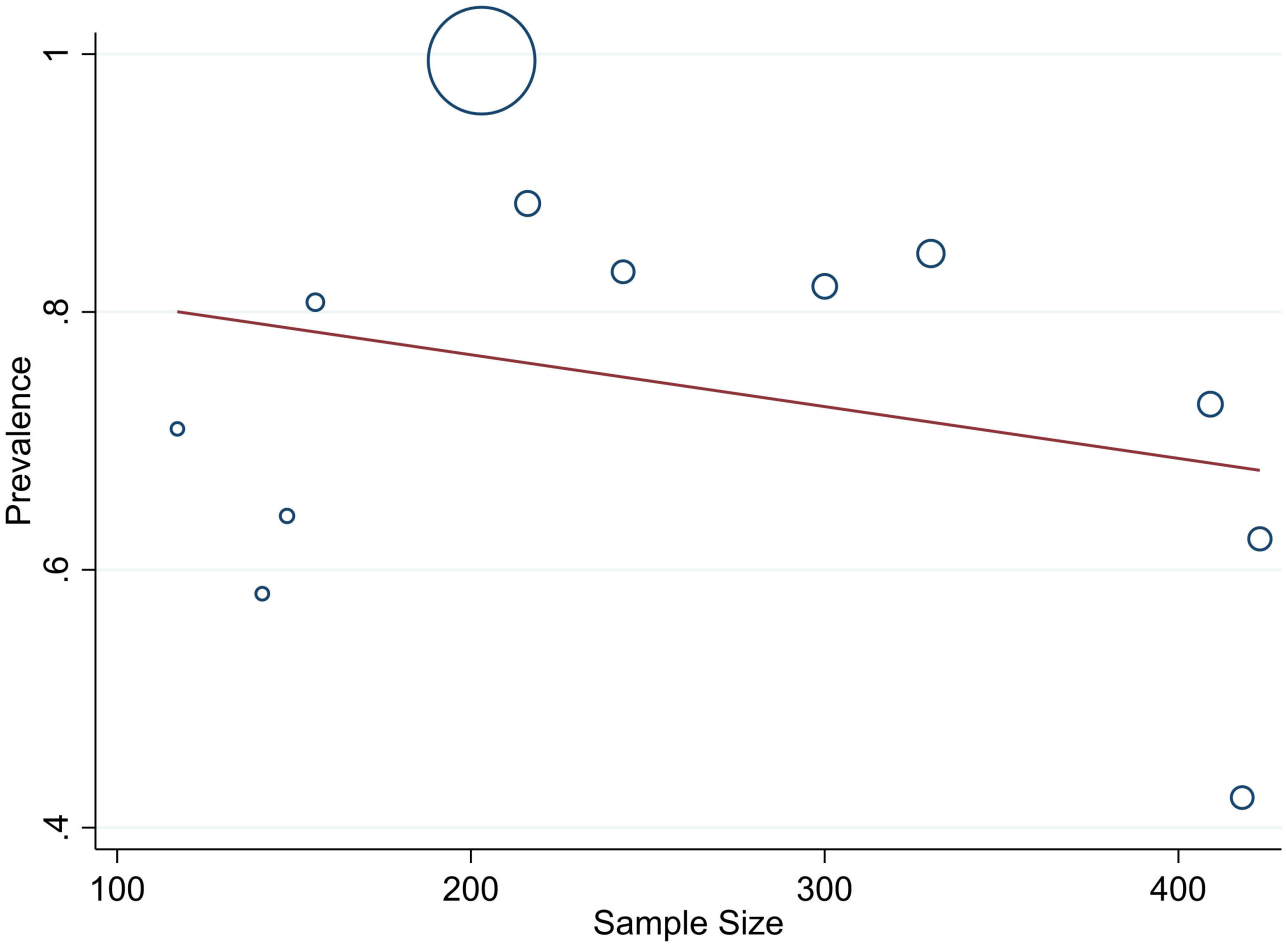
Univariate Meta-regression of prevalence of unmet drug-related need and sample size.

**Figure 9:**
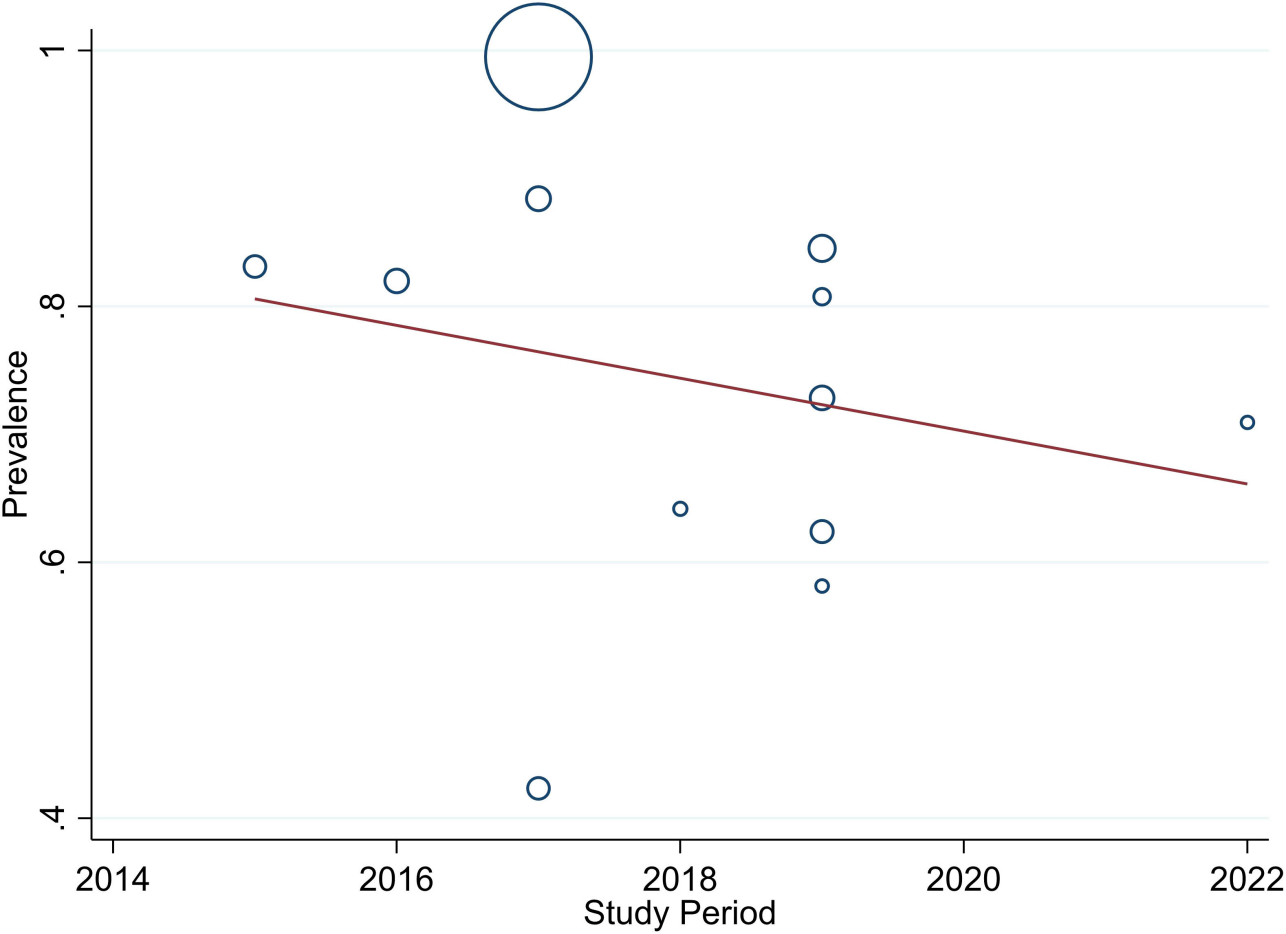
Univariate Meta-regression of prevalence of unmet drug-related need and study periods.

The studies incorporated into the analysis exhibited substantial heterogeneity (I^2^ = 99.0%; p value < 0.001), and the application of a weighted inverse variance random-effects model did not adequately address this variability. To further explore and understand the heterogeneity, we employed a forest plot (**Figure 3**) for subjective assessment and conducted subgroup analysis, along with univariate meta-regression utilizing sample size and study period as variables with respective (tua^2^ = 0.2375 and P=0355) and (tau^2^ = 0.2364 and P=0.452) (**Figures 8**, **9**) which revealed that they are not source of heterogeneity.

### Publication bias

**Figure 10:**
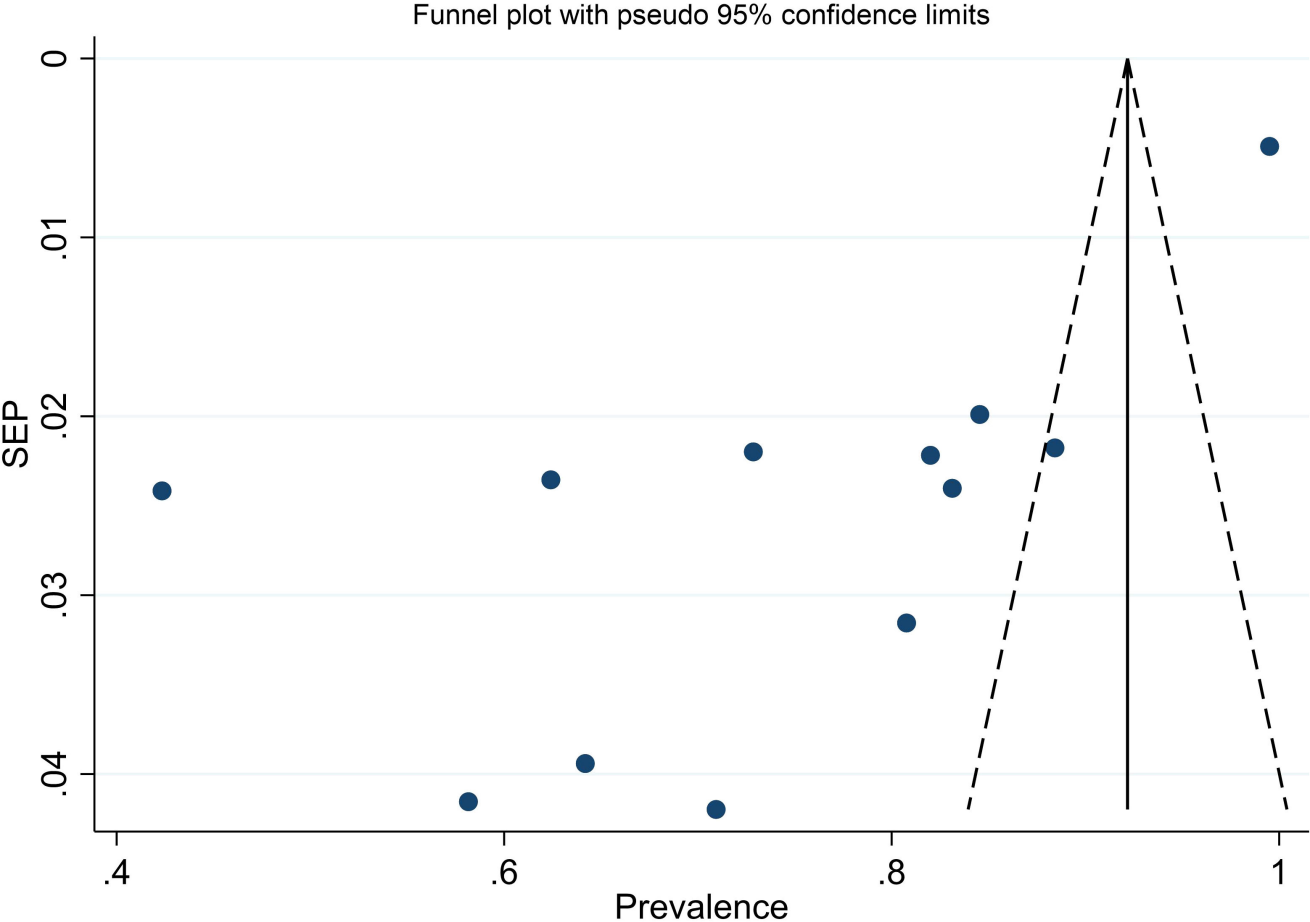
Funnel plot of prevalence of unmet drug-related needs of DM patients in Ethiopia.

We evaluated publication bias by subjectively examining the funnel plot (**Figure 10**) and conducting Egger’s regression test, which yielded a p-value of <0.001, signaling the need for further investigation into publication bias. Subsequent trim and fill analysis was executed, indicating that no studies needed to be added or removed. This analysis ultimately confirmed the absence of missed small studies.

### Sensitivity analysis

A sensitivity analysis was conducted on the prevalence of unmet drug-related needs utilizing a random effects model (**Table 3**). Each of the excluded studies demonstrated slight instability in the aggregated prevalence of unmet drug-related needs in Ethiopia.

**Table 3:** Sensitivity analysis of studies included for estimation of pooled prevalence of unmet drug-related needs.

Study Omitted	Estimate	[95% Conf. Interval]	
Koyra et al	.73381746	.61187404	.85576093
Yimama et al	.73482263	.61222136	.85742396
Ayele et al	.7190181	.63202333	.80601287
Abdulmalik et al	.75098777	.63479453	.867181
Argaw,et al	.72891068	.60497195	.85284942
Demoz et al	.7718274	.67653024	.86712456
Mechessa et al	.75630426	.64126241	.87134606
Kefale et al	.7529273	.63946962	.86638504
Sheleme et al	.7324369	.60767567	.85719806
Belayneh et al	.73607904	.61657888	.8555792
Negash et al	.74325025	.62386566	.86263484
Kahssay et al.	.74494278	.62763917	.86224639
**Combined**	**.7420593**	**.63004919**	**.85406942**

The values in bold represent the aggregated effect size of unmet drug-related needs across the studies or papers listed.

### Contributing factors associated with unmet drug-related needs of DM patients

In our comprehensive review, we have examined factors that contribute to unmet drug-related needs in patients with DM. Commonly identified factors include the use of multiple medications, advanced age, the presence of comorbidities, and an extended duration of illness. Conversely, patients on insulin monotherapy, living out of the capital (Addis Ababa) and those with a history of hospitalization were found to have a lower risk of experiencing unmet drug-related needs (**Table 4**).

**Table 4:** List of contributing factors for unmet drug-related needs of patients with DM in Ethiopia.

Studies	Study Participants	Factors Identified	AOR(95% CI)	P-Value
**Demoz et al**	418	Type 2 DM	5.62(1.21–26.04)	0.027
		Living out of Addis	0.30(0.12–0.73)	0.008
		Female	2.31(1.30–4.12)	0.004
		Ever married	2.58(1.23–5.48	0.013
		Comorbidity ‗ 3	3.61(1.19–10.96)	0.023
		Insulin(s) alone therapy	0.57(0.34–0.96)	0.034
		Low adherence	5.26(2.51–11.04)	<0.001
**Kahssay et al.**	117	Farmer occupation	3.564(1.12–11.38)	0.03
		Four or more comorbidity	1.95(0.9–3.76)	0.02
**Sheleme et al.**	330	Diabetes duration ‗7 yrs.	2.019 (1.059, 3.850)	0.033
		Presence of Comorbidity	2.333 (1.182, 4.604)	0.015
**Yimama et al**	309	Age(41–60)	6.54 (2.58–16.61)	NA
		Age (‗ 60)	5.1 (1.95–13.3)	NA
		Presence of Comorbidity	3.01 (1.11–8.16)	NA
**Negash et al**	409	Primary school education level	2.94(1.25–6.91)	NA
		Self-insured	2.27(1.08–4.77)	NA
**Kefale et al.**	423	Age (45–65)	2.55 (1.28–5.11)	0.008
		Low Family income	4.64 (1.44–14.99)	0.010
		Presence of comorbidities	9.19 (4.78–17.69)	<0.001
		Number of medications ‗5	2.84 (1.72–4.70)	0.001
**Koyra et al**	243	Polypharmacy	3.311(1.366-30.329)	NA
		History of hospitalization	0.403(0.176-0.925)	NA
		Presence of comorbidity	7.004(1.285-18.194)	NA
		Age (45-54)	4.851(1.129-20.853)	NA
		Age (55-64)	6.878(1.930-24.511)	NA
		Age (‗65)	9.079(2.213-37.241)	NA
**Argaw et al.**	216	Age (46-59)	3.537(1.207-10.364)	0.021
		Age(≥ 60)	5.467(1.599-18.693)	0.007
**Belayneh et al**	156	Age (≥45)	5.59 (1.38–20.64)	0.016
		Presence of comorbidity	3.22 (1.75–13.47)	0.014
**Mechessa et al**	141	Poly pharmacy	6.27(1.67–23.52)	0.006
		Duration of DM (6-10 yrs)	3.89(1.52–9.95)	0.004
		Duration of DM(‗10 yrs)	4.36(1.44–13.22)	0.009
		Presence of comorbidity	4.12(1.71–9.91)	0.002

NA, Not available.

## Discussion

This is a pioneer systematic review and meta-analysis study done in Ethiopia that examined the overall pooled estimates of prevalence of unmet drug related need of diabetic patients alongside with the frequently encountered types of drug therapy problems. In addition, this study explored multiple factors that are either positively or negatively associated with unmet drug related needs of diabetic patients in Ethiopia.

The pooled prevalence of unmet drug-related needs of diabetic patients in Ethiopia was 74%. This eye-opening finding is higher than a general prevalence of drug therapy problems in Ethiopian public health institutions 69.4% (51). The disparities between the prevalence rates in specific diabetic populations compared to the general public health institutions shed light on the need for targeted interventions. Tailored initiatives should be implemented to address the unique challenges faced by diabetic patients, aiming to improve medication accessibility, affordability, and overall healthcare delivery. Finding from this meta-analysis is lower from a report of recent global systematic review study (52) pooled from 20 observational studies done on hospitalized type-2 diabetic patients that reported an overall prevalence of 7% to 94% of drug related problems (DRPs). The incongruency could be possibly ascribed to difference in characteristics of study population, where ours pooled studies that were conducted on all types of DM patients who are both ambulatory and/or hospitalized while the ecumenical study was done only in type-2 DM patients who are hospitalized in medical wards. Besides, it could also be linked to variation in methods (chart reviews, incident reports, surveys, direct observation, interviews) implemented to identify DRPs in the included studies as each method is endowed with varying potential and rate of detecting DRPs. In this study, the pooled mean of unmet drug-related need per patient in diabetic patients in Ethiopia was 1.45. This finding sheds light on the prevalent challenges in meeting the medication requirements of diabetic individuals in the Ethiopian setting which underscores the importance of addressing the gaps in drug therapy to enhance the overall management and outcomes for patients with diabetes.

Concerning the specific type of drug therapy problem attested, patients with additional drug therapy needs exhibited the highest proportion (25%) while high dose drug therapy problem displayed a relatively lower proportion (4%). This could be an indicative of lack of adherence to standard treatment guideline, uncoordinated work between prescribers and clinical pharmacists, and poor patient counseling service. In view of our finding, the result of a similar study (52) done elsewhere demonstrated that the most common DRPs experienced by type-2 DM patients were drug-drug interactions (DDIs) [29% to 94%], adverse drug reactions (ADRs) [46.4%], therapeutic effectiveness problems [50.6%], and inappropriate medication use [55.2%]. However, from a theoretical perspective, drug-drug interaction is not regarded as a specific type of DRP rather a cause for most types of DRPs such as dose too high, dose too low, ADRs, and ineffective drug therapy problem. By following a systematic approach to identify and discontinue medications in which potential harm preponderate the benefit and medications with ill-defined benefit, it is possible to abridge exposure to DRPs.

The sub-group analysis of this study indicated that about 79% of unmet drug-related need were reported in type-2 DM patients, 82% were accounted from observational studies that employed retrospective study designs, and 82% were from studies conducted in Harar, Ethiopia. So far, comprehensive reviews have not been done anywhere in relation to study setting, and study designs. However, in terms of study characteristics, this finding is in conformity to a nascent similar study which found a 7% to 94% unmet drug related needs solely in type-2 DM patients (52).

According to this study, the use of multiple medications, advanced age, the presence of comorbidities, and an extended duration of illness were the most frequently reported factors associated with unmet drug related need of patients with diabetes while taking insulin monotherapy and having history of hospitalization were found to have a lower risk of facing unmet drug-related needs. Consonant to our study, a nascent systematic review and meta-analysis study (52) done solely on hospitalized type-2 DM patients unveiled that the presence of comorbidities and ingestion of antihypertensive medications enhances exposure to DRPs, possibly due to increasing multiple drug therapy and drug-drug interaction. Thus, to successfully prevent DRPs early in DM patients, thorough review of patients’ medical illness and treatment protocols should be accomplished to encourage appropriate prescribing of medications. A qualitative survey compiled from Brazil, China, and Russia on unmet needs of type-2 DM patients reported more information and support regarding food and diet (36%), designing novel solution to monitor blood glucose levels such as non-invasive devices (36%) and boosting awareness about type-2 DM and its treatment protocols (26%) (53). These recommendations help to fulfil the drug related needs of diabetic patients and thereby decrease the occurrence of both actual and potential drug related problems.

One crucial aspect that emerged during our analysis was the substantial heterogeneity among the included studies, as indicated by a high I^2^ value of 99.0% and a p-value less than 0.001. The utilization of a weighted inverse variance random-effects model did not adequately address this variability. In our effort to better understand and address the heterogeneity, we employed a forest plot for subjective assessment, revealing the diverse nature of the individual study estimates. To further explore the potential sources of heterogeneity, we conducted a subgroup analysis and univariate meta-regression, considering sample size and study period as variables. The results, with respective tau-squared values of 0.2375 (P=0.355) and 0.2364 (P=0.452), suggested that neither sample size nor study period significantly contributed to the observed heterogeneity. This implies that other factors, not explicitly explored in this study, might be influencing the variations among the included studies.

## Strength and limitations of the study

This pioneering research in the Ethiopian context marks the first attempt to establish pooled prevalence estimates of unmet drug-related needs among diabetic patients, alongside an exploration of contributing factors. It stands out as a groundbreaking global study that comprehensively reviews existing literature on drug-related needs, encompassing both ambulatory and hospitalized diabetic patients across various types of diabetes. However, it is crucial to acknowledge certain limitations. The study reveals significant heterogeneity between the included studies, as indicated by the I^2^ statistic. Additionally, the presence of publication bias is noteworthy constraints within the scope of this research.

## Conclusion and recommendations

A considerable proportion of diabetic patients in Ethiopia face significant unmet drug-related needs, with the most prevalent issue being ineffective drug therapy. Factors such as the use of multiple medications, advanced age, the presence of comorbidities, and prolonged illness duration contribute to an elevated risk of experiencing unmet drug-related needs among diabetic patients. Consequently, healthcare professionals, especially prescribers, should be mindful of these factors to proactively prevent unmet drug-related needs in individuals with diabetes. To develop practical strategies for identifying, addressing, and preventing drug-related problems, future studies in this area are imperative. Clinical pharmacists stationed in medical wards and outpatient departments can play a vital role by efficiently identifying, classifying, and resolving drug-related problems through collaborative efforts with other healthcare professionals. This collaborative approach aims to enhance patient clinical outcomes and overall diabetes management.

## Data availability statement

The original contributions presented in the study are included in the article/**Supplementary Material**. Further inquiries can be directed to the corresponding authors.

## Author contributions

MG: Conceptualization, Formal analysis, Methodology, Supervision, Validation, Writing – original draft, Writing – review & editing. NT: Conceptualization, Data curation, Investigation, Methodology, Writing – original draft, Writing – review & editing. TS: Conceptualization, Data curation, Methodology, Writing – original draft, Writing – review & editing. MD: Conceptualization, Methodology, Writing – original draft, Writing – review & editing. TW: Conceptualization, Data curation, Formal analysis, Writing – original draft, Writing – review & editing. YB: Data curation, Methodology, Writing – original draft, Writing – review & editing. AB: Methodology, Supervision, Writing – original draft, Writing – review & editing. MH: Conceptualization, Data curation, Investigation, Methodology, Writing – original draft, Writing – review & editing.
