# Supplementary material for: The unmet drug-related needs of patients with diabetes in Ethiopia: a systematic review and meta-analysis
Source: Front Endocrinol (Lausanne). 2024 May 30;15:1399944. doi: 10.3389/fendo.2024.1399944 (PMC11169802; doi:10.3389/fendo.2024.1399944)
Supplement: Supplementary file 1 [file Table_1.docx]

| **Author** | **year** | **Q1** | **Q2** | **Q3** | **Q4** | **Q5** | **Q6** | **Q7** | **Q8** | **Q9** | **Total**  **Score** | **Status** |
| --- | --- | --- | --- | --- | --- | --- | --- | --- | --- | --- | --- | --- |
| **Negash** **et al** | **2021** | **Y** | **Y** | **Y** | **Y** | **Y** | **Y** | **Y** | **Y** | **Y** | **9/9** | **Included** |
| **Mechessa et al** | **2020** | **N** | **N** | **N** | **Y** | **Y** | **N** | **Y** | **Y** | **Y** | **6/9** | **Included** |
| **Yimama et al** | **2018** | **N** | **N** | **Y** | **Y** | **Y** | **Y** | **N** | **Y** | **Y** | **6/9** | **Included** |
| **Sheleme et al** | **2021** | **N** | **N** | **Y** | **Y** | **Y** | **Y** | **Y** | **Y** | **Y** | **7/9** | **Included** |
| **Demoz et al** | **2019** | **N** | **U** | **Y** | **Y** | **Y** | **Y** | **Y** | **Y** | **Y** | **7/9** | **Included** |
| **Ayele et al** | **2018** | **N** | **N** | **N** | **Y** | **Y** | **Y** | **Y** | **N** | **Y** | **5/9** | **Included** |
| **Abdulmalik et al** | **2019** | **N** | **Y** | **N** | **Y** | **Y** | **Y** | **Y** | **N** | **Y** | **6/9** | **Included** |
| **Kahssay et al** | **2023** | **Y** | **Y** | **N** | **Y** | **Y** | **Y** | **Y** | **Y** | **Y** | **8/9** | **Included** |
| **Belayneh et al** | **2021** | **N** | **N** | **Y** | **Y** | **Y** | **Y** | **N** | **Y** | **Y** | **6/9** | **Included** |
| **Argaw et al** | **2020** | **N** | **N** | **N** | **Y** | **Y** | **Y** | **N** | **Y** | **Y** | **5/9** | **Included** |
| **Koyra et al** | **2017** | **N** | **N** | **N** | **Y** | **Y** | **Y** | **Y** | **Y** | **Y** | **6/9** | **Included** |
| **Kefale et al** | **2020** | **N** | **N** | **Y** | **Y** | **Y** | **Y** | **Y** | **Y** | **Y** | **7/9** | **Included** |

**Quality assessment of studies included for meta-analysis Using JBI Checklist**

***Y: Yes, N:No***
